# Trends in incidence and mortality of acute and chronic leukaemias in Brazil: challenges and advances over three decades

**DOI:** 10.3332/ecancer.2026.2106

**Published:** 2026-04-03

**Authors:** Kaio Mota-Ribeiro, Maria P Curado, Betine P M Iser, Mohsen Naghavi, Deborah C Malta, Max M Oliveira

**Affiliations:** 1 Institute of Tropical Pathology and Public Health, Federal University of Goiás, Rua 235, Setor Universitário, Goiânia, GO 74605-050, Brazil; 2 Ribeirão Preto Medical School, University of São Paulo, Av Bandeirantes 3900, Ribeirão Preto, SP 14049-900, Brazil; 3 A.C. Camargo Cancer Center, Rua Professor Antônio Prudente 211, Liberdade, São Paulo, SP 01509-010, Brazil; 4 University of Southern Santa Catarina, Av José Acácio Moreira 787, Tubarão, SC 88704-900, Brazil; 5 Institute for Health Metrics and Evaluation, School of Medicine, University of Washington, 3980 15th Ave NE, Seattle, WA 98195, USA; 6 School of Nursing, Federal University of Minas Gerais, Av Professor Alfredo Balena 190, Belo Horizonte, MG 30130-100, Brazil; a https://orcid.org/0000-0001-7945-9022; b https://orcid.org/0000-0001-8172-2483; c https://orcid.org/0000-0001-6061-2541; d https://orcid.org/0000-0002-6209-1513; e https://orcid.org/0000-0002-8214-5734; f https://orcid.org/0000-0002-0804-5145

**Keywords:** cancer, epidemiology, haematologic oncology, public health

## Abstract

Leukaemias are malignant neoplasms that affect the bone marrow, compromising the production of functional blood cells. In Brazil, leukaemia is the 13th most frequent cancer and the most common among individuals up to 20 years old. This study analysed the incidence and mortality of acute and chronic myeloid and lymphoid leukaemias in Brazil from 1990 to 2021, using adjusted and estimated data from the Global Burden of Diseases Study 2021. This is an ecological time-series study with age-standardised rates, expressed per 100,000 inhabitants. Trends were assessed using Joinpoint regression, considering the average annual percentage change. The results indicated an increase in the incidence of chronic lymphocytic leukaemia (CLL) and Acute Myeloid Leukaemia in males, as well as an increase in AML-related mortality in both sexes. On the other hand, a reduction in the incidence and mortality of acute lymphocytic leukaemia (ALL) and chronic myeloid leukaemia (CML) was observed in both sexes. ALL-related mortality also decreased in females, whereas CLL-related mortality increased in males. According to age group and sex, varied trends were observed for these neoplasms. These findings highlight the need for further studies on haematologic neoplasms to inform public health policies, foster research on targeted therapies and promote advancements in the management of these diseases in Brazil.

## Introduction

Leukaemias are haematologic neoplasms that affect blood cells and bone marrow. Their primary characteristic is the accumulation of immature and/or dysfunctional cells, which replace normal and functional cells in the body, triggering physiological disturbances in patients [1]. Globally, leukaemias rank between the 8th and 12th positions in cancer mortality statistics, with variations across countries [2]. Although incidence is higher in urbanised areas, leukaemias affect populations in all regions and sociodemographic contexts. In low-income countries, morbidity and mortality rates are higher, primarily due to limitations in access to diagnosis and treatment, which are associated with high costs and the need for advanced medical infrastructure [3].

Countries with a high Human Development Index show a higher incidence of leukaemias, a factor partially attributed to population aging and access to early diagnosis, which is closely linked to the quality of healthcare systems [2, 4]. In 2020, leukaemias were responsible for approximately 474,000 new cases and 311,000 deaths worldwide. During the same period, in South America, over 25,000 new cases and 18,000 deaths were recorded [5].

In the United States, an estimated 59,600 new cases and 23,700 deaths from leukaemia occurred in 2023, representing 3% of diagnosed cancer cases and 3.9% of cancer-related deaths in the country [6]. In Brazil, between 2023 and 2025, an annual incidence of approximately 11,540 leukaemia cases is estimated. The disease distribution shows a higher prevalence among men, with 6,250 expected cases per year, compared to 5,290 cases among women, corresponding to an estimated risk of 5.33 cases per 100,000 men and 4.78 cases per 100,000 women [7].

Unlike other types of cancer, leukaemias do not form solid tumours detectable through imaging exams. They encompass various subtypes, which vary according to the progenitor cell and the patient’s age, factors that influence prognosis and therapeutic options [1]. The etiology of leukaemias remains unknown, although documented risk factors include exposure to benzene, pesticides, formaldehyde, radiation and genetic mutations. However, the association between these factors and the development of leukaemia has not yet been fully established [8]. Other potential risk factors include smoking [10], family history of cancer, genetic disorders such as Down syndrome and previous cancer treatments [9].

Leukaemias represent a significant public health challenge in Brazil due to the complexity of diagnosis and treatment, which often require specialised healthcare infrastructure, as well as the high mortality rates. Aiming to inform public policies, this study sought to analyse the incidence and mortality rates of acute myeloid leukaemia (AML), chronic myeloid leukaemia (CML), acute lymphocytic leukaemia (ALL) and chronic lymphocytic leukaemia (CLL) over the past three decades.

## Methods

This study is characterised as an ecological study, with a time-series analysis of incidence and mortality rates related to AML, CML, ALL and CLL in Brazil from 1990 to 2021. Data for the indicators were obtained from the Global Burden of Disease Study 2021, accessed on 27 May, 2024, and available at https://vizhub.healthdata.org/gbd-results/. Age-standardised mortality rates (ASMRs) were calculated per 100,000 inhabitants [11].

The variables included were incidence and mortality rates, disaggregated by sex (male and female) and age group (0–14, 15–29, 30–59, 60 years or older and all ages). Leukaemia classifications were defined by GBD codes, which align with the International Classification of Diseases (ICD): C92.0 (AML), C92.1 (CML), C91.0 (ALL) and C91.1 (CLL) [1, 2, 29].

Population estimates are numerical values aimed at estimating unknown parameters of a population, based on data collected from a representative sample. In the Brazilian context, the GBD 2021 estimates incorporated data from multiple national sources, which can be detailed at https://ghdx.healthdata.org/geography/brazil. Mortality estimates were primarily derived from the Mortality Information System (Sistema de Informações sobre Mortalidade), managed by the Ministry of Health. For incidence estimation, the GBD framework utilised data from Population-Based Cancer Registries (Registros de Câncer de Base Populacional located in major Brazilian cities. These data sources underwent a rigorous standardisation process, which included the redistribution of ‘garbage codes’ (ill-defined or intermediate causes of death) and corrections for underreporting using the CODEm model. This methodology enables the generation of comprehensive estimates even for regions with incomplete direct coverage, and further details can be consulted at https://ghdx.healthdata.org/ [12].

To characterise the epidemiology of leukaemias and guide public policies, incidence and mortality trends were analysed using the average annual percentage change (AAPC) and 95% confidence intervals (95% CIs) for the years 1990 to 2021. The AAPC provides a measure of average annual changes, aiding in identifying increasing or decreasing trends. The analysis was performed using the Joinpoint Regression Program version 5.2.0.0, April 2024, and results with *p* < 0.05 were considered statistically significant [13].

This study followed Brazilian ethical guidelines, using aggregated secondary data without individual identification, in accordance with Resolution No. 674 of 6 May 2022, which exempts ethical approval for studies using public and anonymous data. The study is linked to the project ‘Study of Sociodemographic Differences in the Epidemiology of Neoplasms,’ approved by the Research Ethics Committee of the Clinical Hospital of the Federal University of Goiás (opinion No. 5,249,241).

## Results

### Acute lymphocytic leukaemia

**Figure 1.**
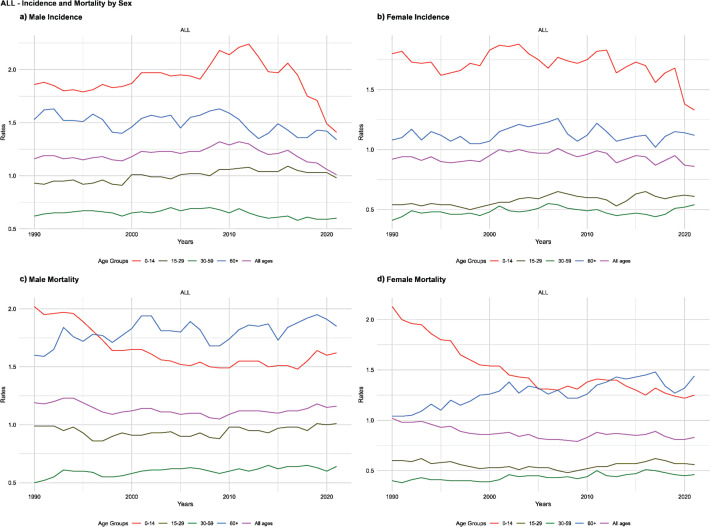
Age-standardised incidence and mortality rates for ALL, by age group and sex. Brazil, 1990–2021. (a) Male incidence. (b) Female incidence. (c) Male mortality. (d) Female mortality.

In Brazil, for males aged 0–14 years, the age-standardised incidence rate (ASIR) of ALL decreased from 1.86 cases per 100,000 inhabitants in 1990 to 1.41 in 2021, with an adjusted AAPC of −0.80% (*p* = 0.001). Among those aged 15–29 years, there was an increase in the incidence rate from 0.93 (1990) to 0.98 (2021), with an AAPC of 0.26% (*p* = 0.032). For individuals aged 30–59 years, the rate decreased from 0.62 to 0.60, with an AAPC of −0.33% (*p* = 0.001). For those over 60 years, the rate decreased from 1.53 to 1.34, with an AAPC of −0.41% (*p* = 0.043). Considering all age groups, the age-standardised rate decreased from 1.16 to 1.01, with an AAPC of −0.50% (*p* = 0.001) (Figure 1, Table 1).

For females aged 0–14 years, the ASIR of ALL decreased from 1.80 in 1990 to 1.33 in 2021, with an AAPC of −1.09% (*p* = 0.001). Among those aged 15–29 years, there was an increase in incidence from 0.54 to 0.61, with an AAPC of 0.28% (*p* = 0.027). For individuals aged 30–59 years, the rate increased from 0.41 to 0.54, with an AAPC of 0.69% (*p* = 0.001). The trend for females over 60 years remained stable, as did the trend for females of all ages (Figure 1, Table 1).

Regarding ALL mortality for males aged 0–14 years, the age-standardised rate decreased from 2.02 in 1990 to 1.62 in 2021, with an AAPC of −0.62% (*p* = 0.001). Among those aged 15–29 years, mortality showed a stable trend. For individuals aged 30–59 years, the rate increased from 0.50 to 0.64, with an AAPC of 0.80% (*p* = 0.001). For those over 60 years, the rate increased from 1.60 to 1.85, with an AAPC of 0.54% (*p* = 0.012). Considering all age groups, the ASMR showed a stable trend, with an AAPC of −0.05% (*p* = 0.284) (Figure 1, Table 1).

For females aged 0–14 years, the ASMR decreased from 2.13 in 1990 to 1.25 in 2021, with an AAPC of −1.73% (*p* = 0.001). Among those aged 15–29 years, there was a decrease in the mortality rate from 0.60 to 0.56, with an AAPC of −0.28% (*p* = 0.003). For individuals aged 30–59 years, the rate increased from 0.40 to 0.46, with an AAPC of 0.65% (*p* = 0.001). For those over 60 years, the rate increased from 1.04 to 1.44, with an AAPC of 1.08% (*p* = 0.001). Considering all age groups, the ASMR decreased from 1.02 to 0.83, with an AAPC of −0.63% (*p* = 0.001) (Figure 1, Table 1).

### Acute myeloid leukaemia

**Figure 2.**
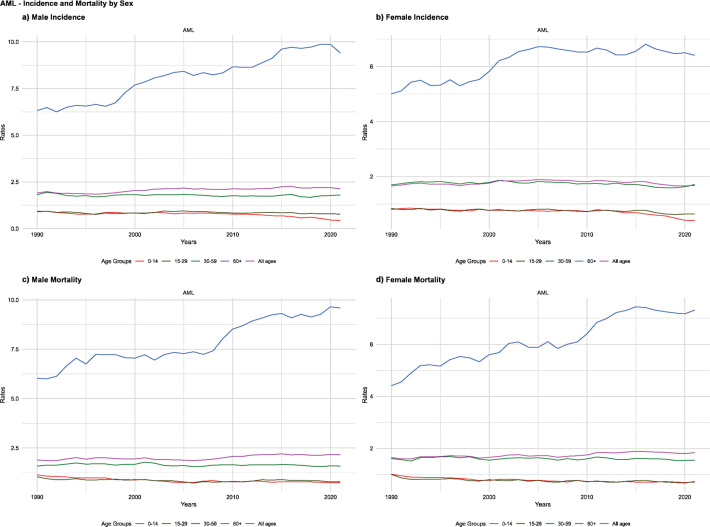
Age-standardised incidence and mortality rates of Acute Myeloid Leukaemia (AML) by age group and sex, Brazil, 1990–2021. (a) Male incidence. (b) Female incidence. (c) Male mortality. (d) Female mortality.

Among individuals aged 0 to 14 years, the incidence of AML showed a significant reduction in both sexes, with the age-standardised rate decreasing from 0.90 to 0.43 in males and from 0.80 to 0.40 in females, with an AAPC of −2.48% and −2.39%, respectively (*p* = 0.001) (Figure 2). A decreasing trend in AML incidence was also observed among adolescents and young adults (15–29 years). In males, the age-standardised rate declined from 0.92 in 1990 to 0.77 in 2021, with an AAPC of −0.61% (*p* = 0.001), while in females, it dropped from 0.84 to 0.64 over the same period, with an AAPC of −0.91% (*p* = 0.006) (Table 1).

Among adults (30–59 years), AML incidence decreased. In males, the age-standardised rate was 1.82 in 1990 and 1.80 in 2021, with an AAPC of −0.13% (*p* = 0.03), whereas in females, the incidence remained stable. Among individuals aged 60 years and older, AML incidence increased. In males, the age-standardised rate rose from 6.32 in 1990 to 9.41 in 2021, with an AAPC of 1.37% (*p* = 0.001), while in females, it increased from 5.01 to 6.41, with an AAPC of 0.87% (*p* = 0.001). Considering all age groups, the ASIR of AML in males increased from 1.91 in 1990 to 2.13 in 2021, with an AAPC of 0.33% (*p* = 0.001), while it remained stable in females (Figure 2, Table 1).

Among individuals aged 0–14 years, AML mortality decreased in both sexes. In 1990, the age-standardised rate was 1.14 for males and 1.01 for females, decreasing to 0.73 and 0.71, respectively, in 2021. The AAPC was −1.29% for males (*p* = 0.001) and −1.10% for females (*p* = 0.001). Among adolescents and young adults (15–29 years), mortality also showed a declining trend. In males, the age-standardised rate decreased from 1.04 in 1990 to 0.78 in 2021, with an AAPC of −0.89% (*p* = 0.001), while in females, it dropped from 1.00 to 0.73, with an AAPC of −1.09% (*p* = 0.001) (Figure 2, Table 1).

Among individuals aged 30–59 years, mortality remained stable in both sexes. However, among those aged 60 years and older, AML mortality increased. In males, the age-standardised rate rose from 6.03 in 1990 to 9.59 in 2021, with an AAPC of 1.55% (*p* = 0.001), while in females, it increased from 4.41 to 7.31, with an AAPC of 1.65% (*p* = 0.001). Across all ages, the age-standardised AML mortality rate in males increased from 1.89 in 1990 to 2.16 in 2021, with an AAPC of 0.47% (*p* = 0.001), while in females, it rose from 1.65 to 1.84, with an AAPC of 0.44% (*p* = 0.001) (Figure 2, Table 1).

### Chronic lymphocytic leukaemia

**Figure 3.**
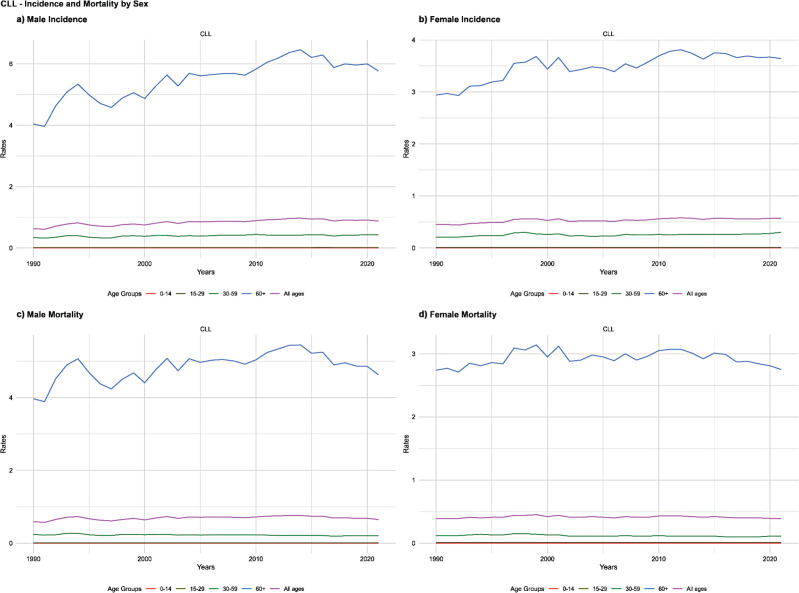
Age-standardised incidence and mortality rates of CLL by age group and sex, Brazil, 1990–2021. (a) Male incidence. (b) Female incidence. (c) Male mortality. (d) Female mortality.

Among adolescents and young adults (15–29 years) of both sexes, the incidence rate trend for CLL remained stable (Figure 3). Among adults (30–59 years), CLL incidence increased. In males, the age-standardised rate rose from 0.34 in 1990 to 0.43 in 2021 (AAPC: 0.72%, *p* = 0.001), while in females, it increased from 0.21 to 0.30 (AAPC: 1.16%, *p* = 0.001). Among individuals aged 60 years and older, CLL incidence also increased. In males, the age-standardised rate rose from 4.04 in 1990 to 5.77 in 2021 (AAPC: 1.26%, *p* = 0.001), while in females, it increased from 2.94 to 3.64 (AAPC: 0.78%, *p* = 0.001). The overall ASIR of CLL in males increased from 0.63 in 1990 to 0.88 in 2021, with an AAPC of 1.25% (*p* = 0.001), while in females, it rose from 0.45 to 0.57, with an AAPC of 0.72% (*p* = 0.001) (Table 1).

Regarding mortality, the trend remained stable among adolescents and young adults. Among adults (30–59 years), CLL mortality decreased. In males, the age-standardised rate declined from 0.24 in 1990 to 0.20 in 2021, with an AAPC of −0.63% (*p* = 0.001), while in females, it decreased from 0.12 to 0.11, with an AAPC of −0.38% (*p* = 0.027). Among individuals aged 60 years and older, mortality increased in males, with the age-standardised rate rising from 3.97 in 1990 to 4.63 in 2021 (AAPC: 0.66%, *p* = 0.001), while it remained stable in females. Across all ages, a rising trend was observed in males, with the age-standardised rate increasing from 0.59 in 1990 to 0.65 in 2021, with an AAPC of 0.49% (*p* = 0.001), whereas in females, mortality remained stable (Table 1).

### Chronic myeloid leukaemia

**Figure 4.**
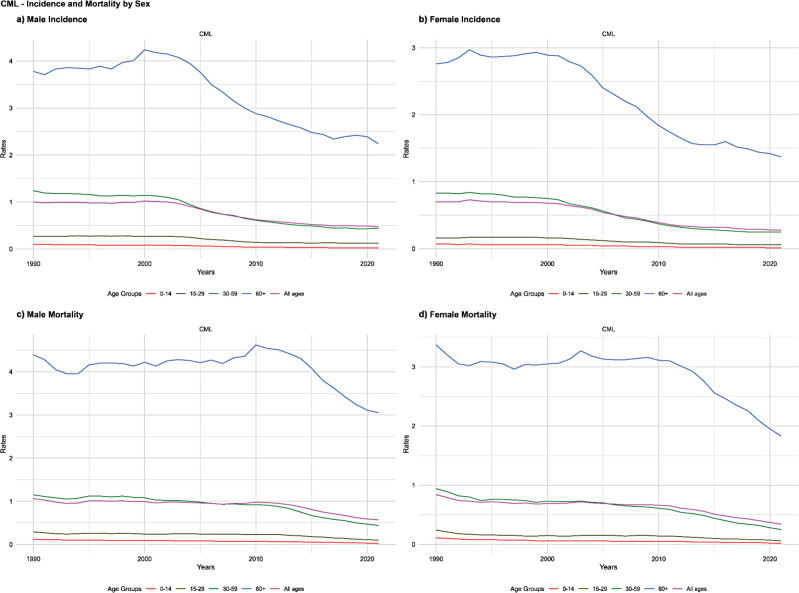
Age-standardised incidence and mortality rates of Chronic Myeloid Leukaemia (CML) by age group and sex, Brazil, 1990–2021. (a) Male incidence. (b) Female incidence. (c) Male mortality. (d) Female mortality.

Among children aged 0–14 years, the incidence rate of CML significantly decreased in both sexes. In boys, the rate declined from 0.10 in 1990 to 0.02 in 2021 (AAPC: −5.31%, *p* = 0.001), while in girls, it decreased from 0.07 to 0.01 (AAPC: −6.31%, *p* = 0.001) (Figure 4). Among adolescents and young adults (15–29 years), CML incidence decreased in males, with the age-standardised rate dropping from 0.27 in 1990 to 0.12 in 2021 (AAPC: −2.62%, *p* = 0.001), and in females, from 0.16 to 0.06 (AAPC: −3.27%, *p* = 0.001) (Table 1).

Among adults (30–59 years), CML incidence declined. In males, the rate decreased from 1.24 in 1990 to 0.44 in 2021 (AAPC: −3.25%, *p* = 0.001), while in females, it dropped from 0.83 to 0.25 (AAPC: −3.79%, *p* = 0.001). Among individuals aged 60 years and older, the incidence rate decreased in males from 3.78 in 1990 to 2.24 in 2021 (AAPC: −1.52%, *p* = 0.001), and in females from 2.76 to 1.37 (AAPC: −2.21%, *p* = 0.001). Considering all age groups, the incidence rate in males declined from 1.00 in 1990 to 0.47 in 2021 (AAPC: −2.34%, *p* = 0.001), and in females from 0.70 to 0.28 (AAPC: −3.00%, *p* = 0.001) (Figure 4, Table 1).

Among children aged 0–14 years, CML mortality also decreased in both sexes. In boys, the rate declined from 0.12 in 1990 to 0.03 in 2021 (AAPC: −4.32%, *p* = 0.001), while in girls, it dropped from 0.11 to 0.02 (AAPC: −5.01%, *p* = 0.001). Among adolescents and young adults (15–29 years), CML mortality showed a downward trend as well. In males, the standardised mortality rate fell from 0.29 in 1990 to 0.10 in 2021 (AAPC: −3.37%, *p* = 0.001), while in females, it declined from 0.24 to 0.06 (AAPC: −4.20%, *p* = 0.001) (Figure 4, Table 1).

Among adults (30–59 years), CML mortality decreased. In males, the rate dropped from 1.15 in 1990 to 0.44 in 2021 (AAPC: −3.05%, *p* = 0.001), while in females, it fell from 0.94 to 0.25 (AAPC: −4.09%, *p* = 0.001). Among individuals aged 60 years and older, CML mortality also declined, with the rate decreasing in males from 4.39 in 1990 to 3.05 in 2021 (AAPC: −1.28%, *p* = 0.001) and in females from 3.37 to 1.83 (AAPC: −1.89%, *p* = 0.001). Overall, CML mortality in males decreased from 1.06 in 1990 to 0.57 in 2021 (AAPC: −2.06%, *p* = 0.001), and in females from 0.84 to 0.34 (AAPC: −2.84%, *p* = 0.001) (Figure 4, Table 1).

## Discussion

This study analysed GBD data on leukaemia in Brazil from 1990 to 2021. A reduction in the incidence and mortality of ALL and CML was observed in both sexes, along with an increase in the incidence of CLL and AML in males, as well as an increase in AML mortality in both sexes. According to age group and sex, varying trends in these neoplasms were noted. Except for ALL, the other leukaemias exhibited higher rates among populations aged 60 years and older, with a trend of increase.

The incidence and mortality trends of leukaemias vary globally, reflecting advancements in diagnosis, treatment and early detection, as well as changes in environmental, genetic and healthcare access factors [2, 14–16]. In recent years, an increasing trend in leukaemia incidence has been observed in many regions, likely due to improvements in diagnostic methods and population aging, as age is a significant risk factor for different forms of leukaemia, especially AML and CLL [8].

Both incidence and mortality rates of leukaemia have shown significant variations across age groups and sexes. Among children and adolescents (0–14 years), there has been a general downward trend in both incidence and mortality, reflecting advancements in early diagnosis and more effective treatments. In young adults (15–29 years), incidence trends have varied, but mortality has shown a declining trend, particularly among women. For adults (30–59 years), incidence trends were mixed, but there was an increase in mortality, highlighting the need for more effective treatment strategies. In older adults (60+ years), both incidence and mortality exhibited fluctuations, underscoring the complexity of treatment in this age group and the vulnerability of these individuals to these diseases.

The incidence and mortality rates of ALL vary across age groups and sexes, with a higher prevalence among men [2]. In children and adolescents, incidence and mortality rates have decreased. However, in adults, particularly older individuals, there is a trend of increasing incidence and, in some cases, mortality. This may be associated with population aging and the biological characteristics of leukaemias in these age groups, which present a poorer prognosis and a 5-year survival rate below 40% [17, 18]. Differences between sexes are also notable, with variations in incidence and mortality trends potentially attributed to biological factors [16, 19].

In Brazil, from 1990 to 2021, incidence and mortality rates decreased among children and adolescents, exhibiting variations by age group and sex. In young adults, incidence and mortality rates declined, more markedly among women. Among adults, both incidence and mortality remained stable, indicating a possible stabilisation of AML patterns in this age group. In contrast, among older adults, both incidence and mortality increased, suggesting the need for better management and treatment strategies for this population [20, 21].

The increase in mortality rates among older adults may be associated with lower tolerance to intensive treatments and the aggressiveness of the disease in this age group. While leukaemia risk factors include genetic predisposition, exposure to chemicals, radiation and certain viral infections, these predispositions vary among different leukaemia subtypes, creating a complex interaction between these factors and host biology that influences leukaemia incidence and mortality across different age groups [1, 22].

Thus, the data indicate a significant reduction in AML incidence among children and adolescents of both sexes, whereas older adults (60+ years) experienced an increase in incidence. Mortality also followed a declining trend in children but increased among older adults. These findings suggest that while advancements in treatment have improved outcomes for younger patients, significant challenges persist for older patients [16, 21].

Regarding CML, a general decrease in incidence and mortality was observed in Brazil from 1990 to 2021 across all age groups and both sexes, making it the only type to exhibit this pattern. This trend is particularly notable among children, adolescents and young adults, where the rate reductions were most pronounced. This decline may be attributed to various factors, such as increased disease awareness, access to adequate medical care, improvements in diagnostic and therapeutic methods, advancements in personalised medicine and the use of tyrosine kinase inhibitors, which have revolutionised CML treatment, leading to greater survival and quality of life for patients [23, 24].

However, although incidence and mortality have decreased, CML remains a concern for older adults. The less pronounced reduction in this age group may be attributed to comorbidities associated with aging, which complicate CML management. This highlights the need to develop prevention and treatment strategies specifically for the elderly population, which remains vulnerable to the disease [25].

The incidence of CLL has shown an increase among adults and older adults of both sexes. Mortality, however, has shown a trend of stabilisation or slight decline across all age groups, except for older adults, where a significant increase was observed. These data suggest that improvements in CLL management and healthcare, which have extended survival, may also have contributed to the increased detection of new cases.

CLL is rare in children and adolescents, as the disease generally affects older individuals. Among adults and older adults, CLL incidence has increased in both sexes, with a more pronounced rise among men. This increase may be linked to the aging Brazilian population and improved diagnostic methods that allow for early disease detection. Additionally, environmental and lifestyle factors may contribute to the rising incidence [21, 26].

While mortality data indicate a decline among adults – possibly due to treatment advances – trends among older adults diverged, with rates remaining stable in women but increasing in men. The persistent increase in mortality among elderly males (60+) can be attributed to a confluence of infrastructural, clinical and sociodemographic factors distinct to the Brazilian reality.

First, regarding infrastructure, there is a critical functional distinction between general oncological access and specialised haematological care. Our analysis of the National Registry of Health Establishments reveals that while the network of High Complexity Oncology Assistance Centers (CACONs/UNACONs) has expanded, there is a stark disparity in the availability of services with the specific ‘Haematology Service’ credential (habilitation code 17.08). A significant portion of units in the North, Northeast and Midwest regions are accredited only for general oncology or radiotherapy. Unlike solid tumours, leukaemias require immediate access to complex hemotherapy support and isolation beds. The concentration of these specialised services (~47%) in the Southeast region forces patients from resource-limited areas to travel long distances. For the elderly population, this geographic barrier imposes high indirect costs (transportation, lodging) and physical fatigue, often compromising treatment adherence.

Second, regarding therapeutic access, a significant gap exists between global approvals and availability within the Brazilian Unified Health System (Sistema Único de Saúde). During the study period (1990–2021), novel agents such as Rituximab and BTK inhibitors (e.g., Ibrutinib, Acalabrutinib) were not widely available in the public network for CLL, with Rituximab only being incorporated for this indication in late 2023. Consequently, public patients, often ineligible for intensive chemotherapy due to frailty and comorbidities, were frequently limited to older, less effective alkylating agents or palliative care. Access to newer drugs often requires judicialisation (lawsuits), a slow and uncertain process that delays urgent care.

Finally, sociodemographic and cultural determinants play a decisive role. The current cohort of elderly Brazilians typically possesses lower formal education levels compared to younger generations, a ‘generational educational gap’ that impacts health literacy. Economically, many rely on low-value state pensions and depend on family members for the financial support required to access centralised treatment centers. Furthermore, culturally rooted behaviours in Brazil, addressed by the National Policy for Integral Attention to Men’s Health, deter men from seeking primary care. This leads to diagnoses at advanced stages where disease burden is high and clinical responses are significantly poorer, creating a ‘perfect storm’ for higher mortality in the elderly male population [26, 27].

Despite advances in CLL detection and treatment, a continued need for targeted management and care strategies exists, particularly for the elderly population. The stabilisation of mortality in women and the rising incidence in both sexes underscore the importance of continued investment in research and improvements in healthcare to reduce the burden of CLL in Brazil.

Therefore, ongoing studies are essential to understand better the mechanisms associated with the disease and to develop more effective prevention and treatment strategies. A detailed analysis of incidence and mortality trends is crucial for the development of public health policies and clinical interventions aimed at improving early detection, treatment and supportive care for leukaemia patients in Brazil.

Furthermore, research on new therapies and the expansion of healthcare access are fundamental to continuing the reduction of leukaemia mortality, both in Brazil and worldwide. Therapeutic advancements, improved early diagnosis and enhanced healthcare have also contributed to declining leukaemia mortality. However, it is important to note that these trends are not uniform, as no highly effective treatment exists for all leukaemia types, as seen with CLL and tyrosine kinase inhibitors [28].

Over the past decades, progress has been made in combating leukaemia in Brazil, reflected in decreasing mortality rates and, in some cases, declining incidence. However, the findings also highlight the increasing incidence of some forms of leukaemia among adults and older adults, along with persistent mortality in these groups, indicating the need for more effective prevention strategies and improved clinical management and care for these age groups.

## Limitations

This study presents limitations inherent to the nature of the GBD data, which are based on modeled estimates derived from secondary sources rather than direct primary data collection. While the GBD methodology applies robust statistical corrections – such as the redistribution of ‘garbage codes’ (ill-defined causes of death) and the use of the CODEm tool to address underreporting – the estimates for Brazil face specific challenges regarding data coverage and quality.

A primary limitation lies in the scope of incidence data. Since Population-Based Cancer Registries do not cover the entire national territory, estimates for non-covered areas rely on statistical modeling and Mortality-to-Incidence Ratios derived from covered regions, which may not fully reflect local epidemiological realities. Furthermore, regional disparities in diagnostic capacity affect the specificity of the input data. In remote areas, limited access to essential diagnostic tools, such as immunophenotyping and molecular testing, may lead to the misclassification of leukaemia subtypes in primary records. Consequently, specific molecular distinctions, such as Philadelphia chromosome-positive (Ph+) ALL, could not be analysed separately, as the GBD data aggregation relies on broad ICD codes (C91.0 for ALL and C92.1 for CML) rather than specific molecular profiles [29].

## Conclusion

The temporal analysis of leukaemia incidence and mortality rates in Brazil over three decades reflects significant advances in haematologic oncology, as well as persistent challenges, particularly in the management of leukaemia in specific populations. The increase in the incidence of CLL in both sexes and the mortality rate in males, along with the rise in incidence and mortality rates for AML, highlight the need for special attention to the elderly population and the development of more effective management strategies.

On the other hand, the consistent reduction in incidence and mortality rates for ALL and CML in both sexes, particularly for CML, which showed a significant decrease, demonstrates advances in the diagnosis and treatment of these conditions. These findings support the patterns observed globally and emphasise the importance of public health policies focused on preventive actions, early diagnosis and personalised therapies.

Investments in research and innovation are essential for addressing these malignancies, contributing to reduced mortality and improving the quality of life for patients. The study highlights the relevance of understanding the epidemiological specifics of leukaemia in Brazil to guide efforts in more targeted and equitable public policies.

## List of abbreviations

AAPC: Average annual percentage change ALL: Acute lymphocytic leukaemia, AML: acute myeloid leukaemia, ASIR: age-standardised incidence rate, ASMR: age-standardised mortality rates, CLL: Chronic lymphocytic leukaemia, CML: chronic myeloid leukaemia, SUS: Unified Health System (Sistema Único de Saúde).

## Conflicts of interest

The authors declare that there are no conflicts of interest regarding this work.

## Funding

This research received no specific grant from any funding agency in the public, commercial or not-for-profit sectors.

## Author contributions

KMR: Conceptualisation, Data curation, Formal analysis, Writing – original draft, Writing – review & editing. MMO: Supervision, Methodology, Writing – review & editing. MPC, BPMI, MN, DCM: Writing – review & editing.

## Figures and Tables

**Table 1.** Age−standardized rates and AAPC in incidence and mortality for ALL, AML, CLL, and CML by age group and sex. Brazil, 1990−2021.

Age group	Standardized rate		AAPC	95% CI	*p*−value	Standardized rate		AAPC	95% CI	*p*−value
	1990	2021				1990	2021			
**ALL**										
**Incidence**										
	**Male**					**Female**				
0–14	1.86	1.41	−0.80*	(−1.1; −0.58)	<0.001	1.80	1.33	−1.09*	(−1.37;−0.86)	<0.001
15–29	0.93	0.98	0.26*	(0.03;0.42)	0.032	0.54	0.61	0.28*	(0.05;0.45)	0.027
30–59	0.62	0.60	−0.33*	(−0.47;−0.19)	<0.001	0.41	0.54	0.69*	(0.42;0.96)	0.001
60+	1.53	1.34	−0.41*	(−0.77;−0.04)	0.043	1.08	1.12	0.03	(−0.43;0.47)	0.867
All ages	1.16	1.01	−0.50*	(−0.70;−0.30)	<0.001	0.92	0.86	−0.17	(−0.35;0.00)	0.053
**Mortality**										
0–14	2.02	1.62	−0.62*	(−0.75;−0.47)	<0.001	2.13	1.25	−1.73*	(−1.87;−1.63)	<0.001
15–29	0.99	1.01	−0.06	(−0.21;0.11)	0.446	0.60	0.56	−0.28*	(−0.51;−0.12)	0.003
30–59	0.50	0.64	0.80*	(0.57;1.07)	0.001	0.40	0.46	0.65*	(0.44;0.87)	<0.001
60+	1.60	1.85	0.54*	(0.20;0.84)	0.012	1.04	1.44	1.08*	(0.71;1.29)	<0.001
All ages	1.19	1.16	−0.05	(−0.13;0.06)	0.284	1.02	0.83	−0.63*	(−0.76;−0.53)	<0.001
**AML**										
**Incidence**										
0–14	0.90	0.43	−2.48*	(−2.77;−2.26)	<0.001	0.80	0.40	−2.39*	(−2.69;−2.11)	<0.001
15–29	0.92	0.77	−0.61*	(−0.81;−0.42)	<0.001	0.84	0.64	−0.91*	(−1.3;−0.64)	0.006
30–59	1.82	1.80	−0.13*	(−0.25;−0.02)	0.030	1.70	1.71	−0.11	(−0.37;0.01)	0.078
60+	6.32	9.41	1.37*	(1.20;1.49)	<0.001	5.01	6.41	0.87*	(0.70;1.04)	<0.001
All ages	1.91	2.13	0.33*	(0.22;0.41)	<0.001	1.66	1.68	0.03	(−0.08;0.13)	0.500
**Mortality**										
0–14	1.14	0.73	−1.29*	(−1.59;−0.97)	<0.001	1.01	0.71	−1.10*	(−1.27;−0.91)	<0.001
15–29	1.04	0.78	−0.89*	(−1.10;−0.61)	<0.001	1.00	0.73	−1.09*	(−1.30;−0.67)	<0.001
30–59	1.58	1.58	−0.01	(−0.24;0.17)	0.90	1.61	1.55	−0.10	(−0.22;0.01)	0.079
60+	6.03	9.59	1.55*	(1.41;1.70)	<0.001	4.41	7.31	1.65*	(1.48;1.81)	<0.001
All ages	1.89	2.16	0.47*	(0.37;0.56)	<0.001	1.65	1.84	0.44*	(0.32;0.57)	<0.001
**CLL**										
**Incidence**										
0–14	0.00	0.00	−	−	−	0.00	0.00	−	−	−
15–29	0.01	0.01	0.27	(−0.25;0.81)	0.262	0.01	0.01	−	−	−
30–59	0.34	0.43	0.72*	(0.47;0.98)	<0.001	0.21	0.30	1.16*	(0.95;1.44)	<0.001
60+	4.04	5.77	1.26*	(1.03;1.51)	<0.001	2.94	3.64	0.78*	(0.64;0.90)	<0.001
All ages	0.63	0.88	1.25*	(1.00;1.50)	<0.001	0.45	0.57	0.72*	(0.57;0.94)	<0.001
**Mortality**										
0–14	0.00	0.00	−	−	−	0.00	0.00	−	−	−
15–29	0.01	0.01	−	−	−	0.01	0.01	−	−	−
30–59	0.24	0.20	−0.63*	(−0.87;−0.38)	<0.001	0.12	0.11	−0.38*	(−0.6;−0.06)	0.027
60+	3.97	4.63	0.66*	(0.43;0.91)	<0.001	2.74	2.75	0.10	(−0.04;0.24)	0.128
All ages	0.59	0.65	0.49*	(0.25;0.74)	<0.001	0.39	0.39	0.04	(−0.12;0.17)	0.599
**CML**										
**Incidence**										
0–14	0.10	0.02	−5.31*	(−5.66;−4.94)	<0.001	0.07	0.01	−6.31*	(−7.06;−5.73)	<0.001
15–29	0.27	0.12	−2.62*	(−2.75;−2.5)	<0.001	0.16	0.06	−3.27*	(−3.48;−3.05)	<0.001
30–59	1.24	0.44	−3.25*	(−3.4;−3.14)	<0.001	0.83	0.25	−3.79*	(−3.88;−3.70)	<0.001
60+	3.78	2.24	−1.52*	(−1.63;−1.42)	<0.001	2.76	1.37	−2.21*	(−2.32;−2.11)	<0.001
All ages	1.00	0.47	−2.34*	(−2.41;−2.28)	<0.001	0.70	0.28	−3.00*	(−3.09;−2.90)	<0.001
**Mortality**										
0–14	0.12	0.03	−4.32*	(−4.81;−3.82)	<0.001	0.11	0.02	−5.01*	(−5.5;−4.61)	<0.001
15–29	0.29	0.10	−3.37*	(−3.57;−3.2)	<0.001	0.24	0.06	−4.20*	(−4.40;−3.93)	<0.001
30–59	1.15	0.44	−3.05*	(−3.15;−2.94)	<0.001	0.94	0.25	−4.09*	(−4.22;−3.98)	<0.001
60+	4.39	3.05	−1.28*	(−1.42;−1.07)	<0.001	3.37	1.83	−1.89*	(−1.98;−1.78)	<0.001
All ages	1.06	0.57	−2.06*	(−2.14;−1.98)	<0.001	0.84	0.34	−2.84*	(−2.93;−2.73)	<0.001
